# Comprehensive Insights and Advancements in Gel Catalysts for Electrochemical Energy Conversion

**DOI:** 10.3390/gels10010063

**Published:** 2024-01-15

**Authors:** Gazi A. K. M. Rafiqul Bari, Jae-Ho Jeong

**Affiliations:** School of Mechanical Smart and Industrial Engineering, Gachon University, 1342 Seongnam-daero, Sujeong-gu, Seongnam-si 13120, Gyeonggi-do, Republic of Korea

**Keywords:** gel, electrocatalyst, energy conversion, CO_2_RR, OER, ORR

## Abstract

Continuous worldwide demands for more clean energy urge researchers and engineers to seek various energy applications, including electrocatalytic processes. Traditional energy-active materials, when combined with conducting materials and non-active polymeric materials, inadvertently leading to reduced interaction between their active and conducting components. This results in a drop in active catalytic sites, sluggish kinetics, and compromised mass and electronic transport properties. Furthermore, interaction between these materials could increase degradation products, impeding the efficiency of the catalytic process. Gels appears to be promising candidates to solve these challenges due to their larger specific surface area, three-dimensional hierarchical accommodative porous frameworks for active particles, self-catalytic properties, tunable electronic and electrochemical properties, as well as their inherent stability and cost-effectiveness. This review delves into the strategic design of catalytic gel materials, focusing on their potential in advanced energy conversion and storage technologies. Specific attention is given to catalytic gel material design strategies, exploring fundamental catalytic approaches for energy conversion processes such as the CO_2_ reduction reaction (CO_2_RR), oxygen reduction reaction (ORR), oxygen evolution reaction (OER), and more. This comprehensive review not only addresses current developments but also outlines future research strategies and challenges in the field. Moreover, it provides guidance on overcoming these challenges, ensuring a holistic understanding of catalytic gel materials and their role in advancing energy conversion and storage technologies.

## 1. Introduction

The pressing global need for sustainable and efficient clean energy is paramount in countering the extensive use of fossil fuels and mitigating the adverse impacts of climate risks [1,2]. An electrocatalytic energy conversion system in clean energy processes holds the potential to transform environmental pollutants, such as CO_2_, into valuable and environmentally safe energy compounds. The success of this energy conversion process is crucial to meeting the demands of practical applications, considering factors such as cost-effectiveness, stability, and operational flexibility [3]. Metal–air batteries, fuel cells, and water splitting represent pivotal electrochemical energy conversion systems, encompassing processes such as the CO_2_ reduction reaction (CO_2_RR), oxygen evolution reaction (OER), and oxygen reduction reaction (ORR). Practical application of these processes is hindered by sluggish kinetics, efficiency constraints, and selectivity limitations [4,5,6]. While noble metals exhibit efficient catalytic activity, their scarcity and high costs raise significant concerns regarding real-world applications [7]. Recent advancements in gel materials have addressed these challenges by tuning electronic properties, enhancing catalytic active sites, and modulating morphology [8,9]. These improvements contribute to the optimization of electrochemical processes in energy conversion systems, enabling a reduction or dilution of expensive noble metal utilization. Gel-based electrocatalysts thus serve as a guided approach towards enhancing the efficiency and feasibility of these electrochemical processes [10,11,12].

Gel materials are non-fluid colloidal or polymer networks saturated with liquid, exhibiting characteristics of solid materials as three-dimensional networks form within the liquid phase. Nanostructured gels provide a highly porous architecture with abundant defects, facilitating efficient mass transfer and offering opportunities for composition tunability in the generation of catalytic responsive materials [13,14]. The ease of synthesis and functionalization of gel materials enables the production of hierarchical porous structures with high surface areas capable of hosting various catalysts, thereby enhancing mass transport during electrocatalysis processes [15].

In terms of physicochemical properties, gels present a significant number of active sites for reactant or intermediate species, allowing for adsorption, desorption, activation, and conversion or transformation [8]. The formation of these newly developed gel materials involves physical or chemical interactions between the building blocks themselves or between the building blocks and crosslinking agents. Common forces utilized in the gel formation process include Coulomb forces, π–π interactions, hydrogen bonds, hydrophobic interactions, and covalent bonds [11,12].

To maximize catalytic properties, it is essential to achieve a high density of active sites and provide high accessibility to micro- and nano-architectures while maintaining high conductivity for efficient electrocatalytic activity in practical applications [15]. Gel materials find diverse applications as electrode materials, electrolytes, self-supported current collectors, skeletons of active materials, and 3D binders in the field of energy conversion and storage, such as electrochemical CO_2_ reduction to value-added products, oxygen reduction reactions, and the oxygen evolution reaction in metal–air batteries [16].

Electrocatalysts often face challenges such as self-agglomeration, reduced active sites, unfavorable electron and mass transport, poor electrical conductivity, and the inability to provide well-anchored particles (conventional conductive support). This often leads to issues like peeling off, agglomeration, and dissolution. Gel materials, however, exert highly positive impacts on catalyst design. They enhance design flexibility, guide catalytic pathways, improve catalyst stability, offer cost-effective processing considerations, and contribute significantly fewer environmental impacts. This review article aims to elucidate the fundamental aspects of material design strategies for gel-based catalysts in energy conversion processes, encompassing reactions such as ORR, OER, HER, and CO_2_RR.

## 2. Gel Material Synthesis Process

The synthesis processes for gel materials are categorized into: (i) radical polymerization, (ii) hydrothermal/solvothermal methods, (iii) sol–gel methods, and (iv) ligand substitution. Each method offers unique advantages in tailoring the properties of the resulting gel material for specific applications [8].

### 2.1. Radical Polymerization

**Figure 1:**
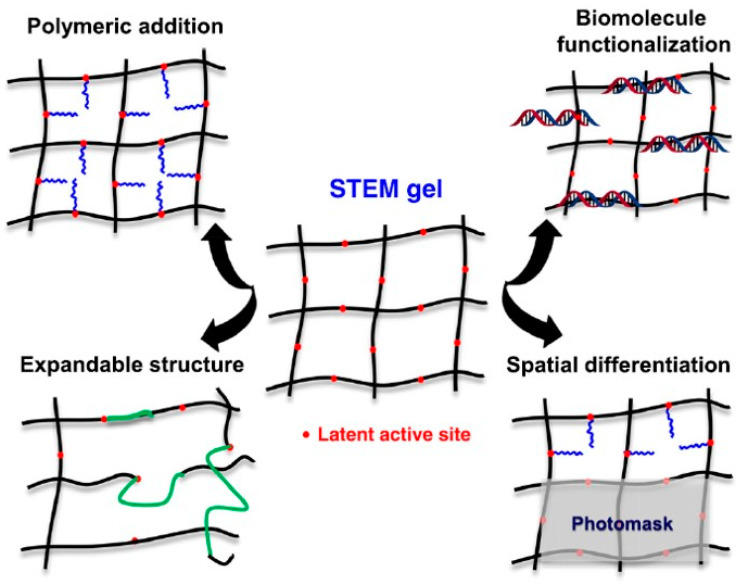
The STEM (structurally tailored and engineered macromolecular) gel concept starts with a basic material (black) and then modifies it through post-synthesis adjustments at latent active sites (radical photo initiator, red). Four examples of these adjustments include polymer side chains, expandable network segments, biomolecule functionalization, and spatial differentiation using a photomask. (Adapted with permission from Ref. [20], copyright 2020 Elsevier Ltd.).

Radical polymerization is employed for the synthesis of organic polymeric gels under mild conditions. Oligomers are used to form polymeric hydrogels through radical polymerization, involving oxidation, deprotonation, or coupling reactions. Free radical polymerization consists of three consecutive steps: initiation, propagation, and termination [17]. Single crosslinker-type polymerization yields hydrogel polymers with numerous hydrophilic groups (Figure 1). Crosslinking agents like acrylic acid, acrylamide, and vinyl acetate are employed to create monomer chemical bonds through UV radiation, temperature, or redox initiator mixing. These agents provide the three-dimensional structure of hydrogels, allowing tuning of the swelling capacity and elastic properties [18,19]. The performance of a hydrogel is modified based on the monomer type, regulation of composition, and modification of various structural crosslinking agents. Improper regulation may result in the production of heterogeneous hydrogel products [20,21]. Additionally, radical polymerization faces technical challenges due to its rapid and difficult-to-control nature.

The exothermic nature of radical polymerization leads to gel effects, releasing a large amount of sudden heat, and posing safety concerns for the process. Gel effects are a significant disadvantage when scaling up polymerization processes on an industrial scale [22].

### 2.2. Hydrothermal/Solvothermal Method

The hydrothermal or solvothermal process involves dissolving heterogeneous reactants in water or organic solvents within a sealed environment under specific temperature and pressure conditions. This method is particularly well-suited for insoluble components and material phases that may be unstable at high temperatures. One notable advantage of this process is its capability to generate new products and product intermediates at lower temperatures compared to alternative methods [23,24]. This process finds application in the synthesis of hierarchical carbon or graphene-based hydrogels. The three-dimensional structures formed result from the combined effects of π–π stacking and hydrophobic interactions. Consequently, a large porous structure is created, facilitating the hosting of active materials within a vast surface area [25,26].

### 2.3. Sol–Gel Method

**Figure 2:**
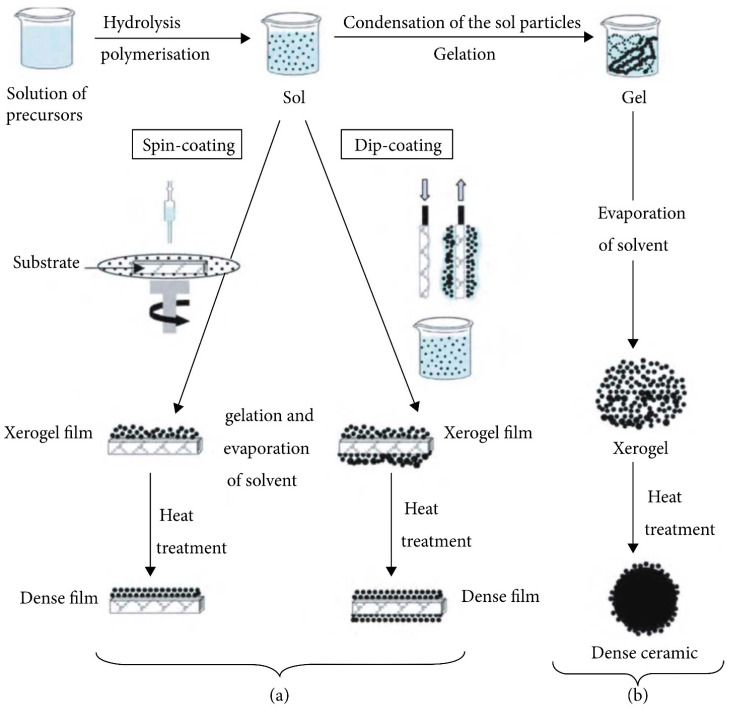
Scheme of sol–gel method: (**a**) xerogel film from sol, and (**b**) xerogel powder from gel. (Adapted with permission from Ref. [27], copyright © 2021 Dmitry Bokov et al.).

In the sol–gel method, precursors undergo a complete transformation into sols at low temperatures. The sol–gel method enables homogeneous doping at the nanoscale, allowing for precise control over a material’s structure (Figure 2). This results in high purity and a uniform distribution of particle size. The cost-effectiveness of this process contributes to its commercial implications and production feasibility. The sol–gel method is particularly adapted for the production of highly chemically active inorganic hydrogels [27,28,29].

The sol–gel process involves the solution of precursors through hydrolysis and partial condensation, leading to the formation of sols. Subsequently, polycondensation of the hydrolyzed precursors results in the formation of the gel product [30,31]. During the drying process, solvent evaporation causes the collapse of the porous network, producing a dense xerogel. On the other hand, supercritical drying leads to the formation of aerogel. Finally, calcination contributes to the production of stable products. The material’s properties can be modified by controlling various parameters in the sol–gel process, including: (i) precursor types, (ii) concentration, (iii) solvent nature, (iv) pH, (v) concentrations of additives (surfactants, structure-directing agents, catalysts), (vi) heat treatment (pre and post treatment), and (vii) aging time. In the sol–gel process, three main sequential reaction steps occur: hydrolysis of the alkoxy group, condensation of two –OH-containing species, and mixed condensation involving the –OH group- and alkoxy group-containing species (Equations (1)–(3)) [32,33,34].

≡M–OR + HOH → ≡M–OH + ROH,(1)

≡M–OH + ≡M–OH → ≡M–O–M + HOH,(2)

≡M–OH + ≡M–OR → ≡M–O–M + ROH,(3)

### 2.4. Ligand Substitution

**Figure 3:**
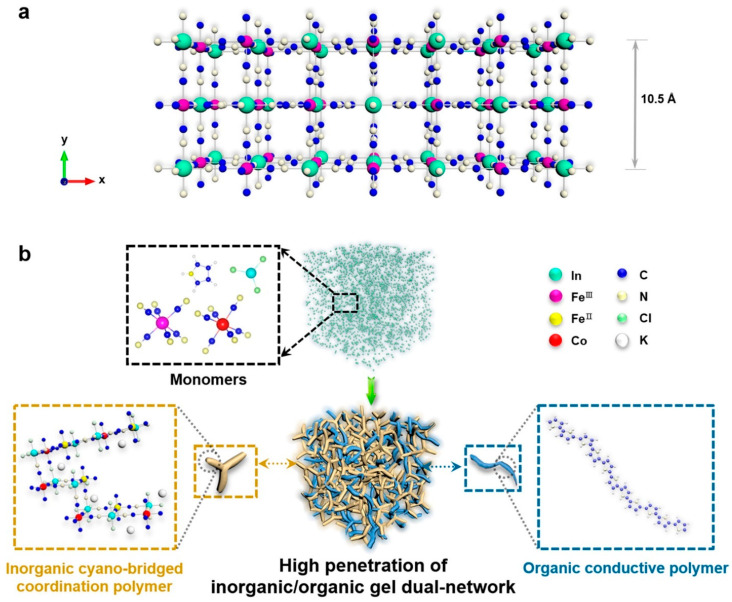
(**a**) Crystal structure of InFe-cyano-bridged coordination polymer. (**b**) Schematic synthesis of hybrid organic–inorganic dual-network gels. (Adapted with permission from Ref. [35], copyright © 2019 American Chemical Society).

The ligand substitution process involves synthesizing coordination-substituted polymers through the polymerization between cyanometallates (e.g., Fe(CN)_6_^4−^, Co(CN)_6_^3−^) and chlorometallates (e.g., InCl_3_, SnCl_4_, RuCl_3_, PdCl_4_^2−^, etc.). Cyanogels are heterometallic gels formed by incorporating different monometallic gels with M and M’ through cyano groups (M–N≡C–M′) (Figure 3). Cyano-bridged coordination polymers represent a type of ligand-substituted gel formation, where alternating arrangements of the main group metal and transition metal are interconnected by the cyano group of the ligand [35,36]. Catalysts of this nature are known for their excellent electrocatalytic properties. Their attractive electrocatalytic properties result from the presence of sufficiently coordinated water and lattice vacancies. The incorporation of multiple metals is homogeneously dispersed, imparting hydrophilic properties. A 3D porous structure with hydrophilic properties facilitates the penetration of electrolytes, enhancing the overall electrocatalytic properties, such as OER [37]. The highly penetrated conductive polymeric gel in an inorganic polymer gel provides excellent and efficient mass and charge transfer ability, significantly contributing to potential energy storage and conversion processes [15,35].

## 3. Gel Classifications

Polymeric gels offer a three-dimensional porous network structure, where the hydrophilic component facilitates water absorption within the structure. Simultaneously, the organic component resists dissolution in water due to crosslinking among the polymeric chains. Gels are categorized based on various factors, including source/origin, polymeric composition, structure/configuration, response to stimuli, durability, network electrical charge, and the presence of crosslinking, air, or water components in the structure. This discussion focuses on two main categories of gels: hydrogels and aerogels. The subsequent exploration delves into their applications and fundamental properties.

### 3.1. Hydrogels

**Figure 4:**
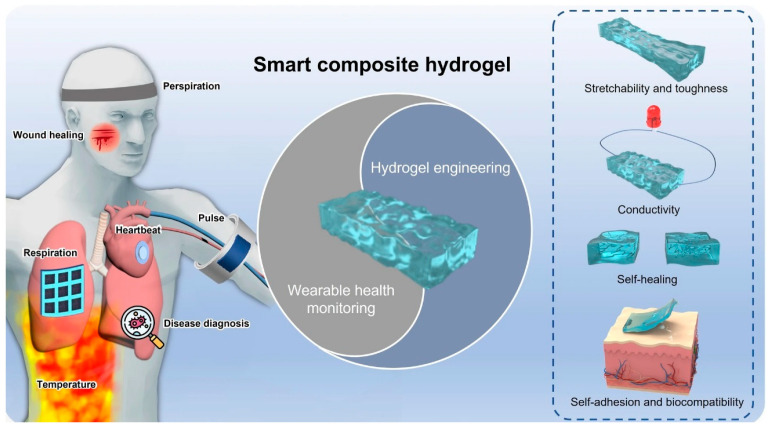
Schematic smart and advanced composite hydrogels for wearable disease monitoring systems. (Adapted with permission from Ref. [44], copyright © 2023 Li et al.).

Gelation chemistry serves as a fundamental framework for modulating a diverse array of physicochemical properties within hydrogels. These properties encompass ionic and electronic conductivities, structural flexibility, stretchability, mechanical strength, swelling characteristics, and responsiveness to external stimuli, including pH, temperature, light, pressure, magnetic fields, and electrical fields [38,39]. Consequently, this versatility broadens the spectrum of applications in the fields of energy conversion and storage, extending to supercapacitors, batteries, fuel cells, electrocatalysts, wearable electronic devices, solar desalination, and water purification [40]. Hydrogels are designed with variable functionalities tailored for specific applications, such as self-healing conductive hydrogels, double-network conductive hydrogels, stimuli-responsive conductive hydrogels, and gamma radiation-induced conductive hydrogels. Self-healing hydrogels find utility in flexible devices or wearable applications, whether covalently bonded or non-covalently bonded (Figure 4). Covalently bonded hydrogels incorporate imine, hydrazine, borate, and disulfide bonds, while non-covalently bonded hydrogels involve coordination bonds of metals, hydrogen bonding, and host–guest π–π stacking bonds. Self-healing hydrogels exhibit dual characteristics through physical and chemical crosslinking, encompassing hydrogen and ionic bonding [41,42,43].

Organic–inorganic hydrogels, featuring metal ions and counter ions, facilitate electron flow and delocalized electrons, enhancing conductivity across the hydrogel networks. Double-network hydrogels are engineered to incorporate rigid and potentially brittle segments alongside flexible and stretchable components, both interpenetrating each other. This technology finds application in wearable smart clothing and devices, where the delocalized double network imparts conductivity to the polymer [45,46]. Stimuli-responsive hydrogels react to external stimuli such as temperature, electric fields, pH, pressure, and near-infrared light, rendering them versatile materials for diverse applications. Their combination of conductive and responsive properties propels technological advancements, particularly in areas like sensors, biomedicine, and tissue engineering [47,48].

### 3.2. Aerogels

**Figure 5:**
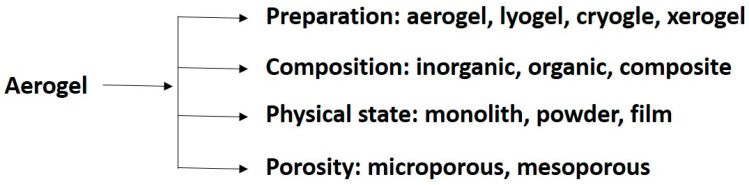
Classification of an aerogel based on preparation, composition, physical state, and structural porosity.

Aerogel is a porous structure with micro-/meso-/macro-pores or nanofibrils, where the dispersed phase is gas. The classification or definition of aerogel is not based on materials or synthesis processes (Figure 5). An aerogel is a structural scaffold soaked with air [49,50]. Aerogel is sometimes referred to as “frozen smoke”, “solid smoke”, “solid air”, or “blue smoke” due to its translucent nature and the way light scatters within the material. Electrocatalytic and electrical properties of aerogels are significant in the field of energy storage, energy conversion, supercapacitors, lightweight optics, and wearable devices. Drying aerogels is a challenging step for scaling them up to an industrial scale [50]. The electrocatalytic activity required for the conversion of their different phases is enhanced due to their 3D structure, which provides a higher surface area, more catalytic active sites, and improved mass and electron transport properties.

**Figure 6:**
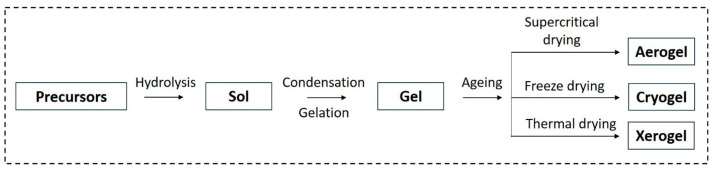
Schematic of aerogel, cryogel, and xerogel production.

In the sol–gel process, the formation of a stable alkoxide or solvated metal from precursors occurs in the solution phase (Figure 6) [51]. Alcohol or oxide bridge networks form the gel through polycondensation or polyesterification, leading to an increase in the viscosity of the solution. The gel undergoes aging until it transforms into a solid mass, resulting in the contraction of the gel network and solvent removal. The aging process is a crucial factor for preventing cracks in the gel. The subsequent drying process determines the desired shape of the gel, resulting in either a highly porous aerogel or a denser xerogel. The solvent evaporation method is employed to remove the solvent and form a wet gel, subsequently producing the xerogel [51,52].

In the case of aerogel drying through a supercritical solvent removal process, a porous network with unhindered shrinkage and low density is formed, maintaining porosity (15–50%) and a high surface area (150–1000 m^2^ g^−1^) within the micro-to-meso-pore range (1–10 nm). The removal of the fluid from the gel tunes the pore morphology, with strong capillary forces generating the xerogel, weak capillary forces creating ambigel, and zero capillary forces producing the aerogel [51].

In the sol–gel method, parameters such as the pH, solvents, and temperature play critical roles. pH control influences the hydrolysis step, leading to the generation of nanoparticles and a gel network. Solvents dissolve the nanoparticles and facilitate their joining together [53]. Temperature governs the formation of gel networks, with a slower formation resulting in a uniform structure and excellent mechanical properties. Acid-based catalysts enhance or accelerate the chemical reaction process, and their tuning can shorten the gel formation process from weeks to days or even weeks to minutes [54].

The supercritical drying process is employed to extract liquid in a controlled manner, ensuring not to cross the liquid–gas boundary during the liquid-to-gas phase transition. In the supercritical phase, high temperature and pressure are applied, where the transition from liquid to gas does not cross any phase boundary but passes through the supercritical region. Carbon dioxide (31.1 °C at 73.9 bar) and nitrous oxide are suitable fluids for supercritical drying, exhibiting similar physical behavior, with the latter demonstrating strong oxidizer behavior in the supercritical state. In a supercritical drying process, alcohol first washes away water, and then alcohol is removed via the liquefied CO_2_. Finally, the liquefied CO_2_ is heated beyond the critical point, resulting in a pressure release, allowing the escape and yielding of the dried products [51,55].

## 4. Gel as an Advanced Catalyst: Challenges and Solutions

Gel-based materials exhibit significant potential as alternative catalysis materials, owing to their versatile utility. Their ease of synthesis, tunable functionalization capability, design flexibility for hierarchical porous structures, low-temperature production, cost-effectiveness, chemical stability, and eco-friendly attributes collectively characterize these gel materials [56]. Gels serve multifunctional roles as electrolytes, electrodes, and binders, leveraging their intrinsic stretchability, flexibility, and bending ability, making them ideal for soft energy devices. Three-dimensional polymer gels efficiently accommodate volume expansion, particularly in managing ion movements during electrochemical processes [57]. A 3D conducting polymer network structure in gels ensures consistent contact as an active electrode, facilitating electronic and ionic conduction with current collector materials. The integration of traditional gels and pristine conducting polymer gels through crosslinking and simultaneous doping enhances their mechanical, electrochemical, and self-healing properties [41,58,59].

In contrast to non-conductive and conventional binder systems, gel materials maintain a consistent morphology of hierarchical pores, facilitating electrolyte and ion diffusions. They provide uniform coating on active particles, eliminating particle delamination and acting as a buffer for volume changes. A robust conducting gel network ensures connected active particles with efficient electron transport, mechanical stability, and chemical stability, contributing to a longer lifespan [57,60,61]. The interconnected nanopores in gels function as nanoscale electrolyte reservoirs, creating ion transport channels around the electrode. Integrating the self-healing properties of polymer gels enables the recovery of electrochemical properties even after mechanical damage to the gel membrane. Abundant functionalization of gel materials with groups such as carboxyl (–COOH), hydroxyl (–OH), carbonyl (–C=O), or amino (–NH_2_) enhances the wettability of hydrogel-based electrolytes, intensifying the contact between the electrode and electrolyte [4,58,59].

Conventional MOF-based materials face challenges in conductivity due to the poor intrinsic conductivity of organic ligands. In contrast, metal–organic gels with hierarchically porous architecture and efficient interconnected conductive pathways to active particles facilitate efficient catalytic activity [56,62]. Porous carbon- and graphene-based catalysts combined with non-conductive binders often result in particle aggregation and volume expansion, leading to fracture and delamination. Gel-based materials, with their volume buffering flexibility, counteract these issues and present a promising alternative for advanced materials in energy conversion technology [57]. 

The synthesis of gel materials presents significant challenges, primarily due to the time-consuming and multi-step nature of the gelation process. Achieving the desired product properties requires careful consideration of various parameters, as deviations within the structure may result in sub-optimal characteristics. Additionally, gel catalysts may face limitations in their activity, leading to performance degradation under harsh operating conditions. The conjugated polymer chain of gels is sensitive to external stress, light, pH, and voltage [48]. Transitioning them to an industrial scale further complicates matters, posing challenges in obtaining uniform and consistent properties for the products. Precise control over textural properties, particularly achieving a homogeneous distribution of pore sizes, proves to be a formidable task. The elastic nature of the gel can cause damage to the hierarchical pore structure, hindering the transport path [57,58].

To address these challenges, simplified design strategies are imperative to scale up the synthesis route and attain uniform desired product properties. The incorporation of a crosslinking agent has shown promise in improving structural integrity, as well as optimizing the elastic nature [63]. Overcoming the sluggish gelation process requires strategic adjustments to various parameters, including reactant concentration ratios, temperature optimization, catalyst concentration adjustments, solvent selection, or the use of solvent mixtures, pH adjustments, and fine-tuning mixing parameters [64,65]. Considering factors such as the incorporation of seed crystals may further alleviate synthesis difficulties. In the pursuit of stability and enhancing the durability of gel materials, encapsulation or the application of a nano-thin protective coating could extend their uniform and consistent performance [66]. Implementing these strategies offers a systematic approach to overcoming the challenges associated with gel material synthesis, paving the way for more efficient and controlled production processes.

### 4.1. Gel Electrocatalysts for CO_2_RR

**Figure 7:**
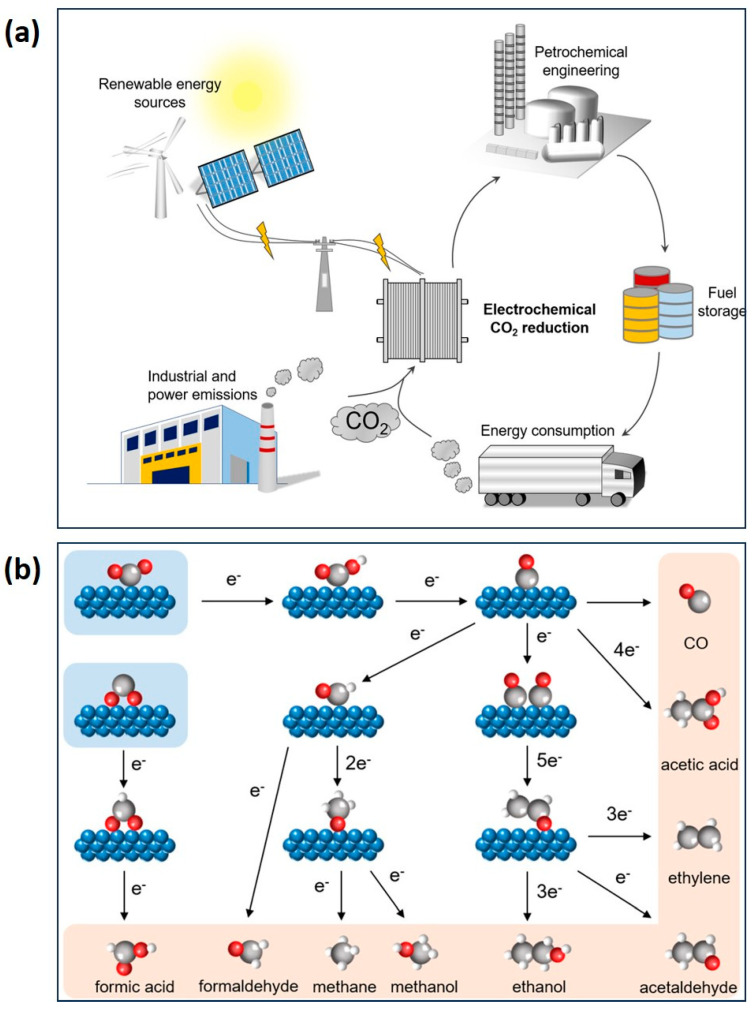
(**a**) Schematic of CO_2_ reduction to produce value-added products using a renewable source, and (**b**) CO_2_RR reaction pathway to different value-added products (blue spheres represent catalysts; gray spheres represent C; red spheres represent O; white spheres represent H). (Adapted with permission from Ref. [69], copyright 2021 Wiley-VCH GmbH).

Continuous emissions of CO_2_ resulting from aggressively higher levels of energy consumption have negative environmental impacts on the Earth. To address the climate challenge and mitigate the concentration of atmospheric CO_2_ (currently at 420 ppm), it is imperative to take action [67,68]. The electrochemical reduction of CO_2_ to produce value-added products such as chemical feedstocks (carbonate, polycarbonate, and polymer building blocks, etc.) and liquid fuels (CH_4_, C_2_H_4_, C_2_H_6_, and CH_3_OH, etc.) is considered a promising approach to fostering a sustainable environment on Earth (Figure 7) [69,70,71,72]. The highly thermodynamically stable CO_2_ molecule necessitates larger overpotentials to achieve CO_2_ radical anions by introducing an electron into the CO_2_ molecules (E^0^ = −1.90 V vs. the standard hydrogen electrode). Additionally, counteracting the competitive hydrogen evolution reaction (HER), which occurs simultaneously with CO_2_ reduction (HER, E^0^ = 0 V vs. the relative hydrogen electrode), poses a challenging task and leads to low selectivity in the desired products [73,74]. Research efforts are currently focused on the design and modification of efficient and economical electrocatalysts for CO_2_ reduction, with the aim of maintaining the selectivity and durability of the catalyst.

**Figure 8:**
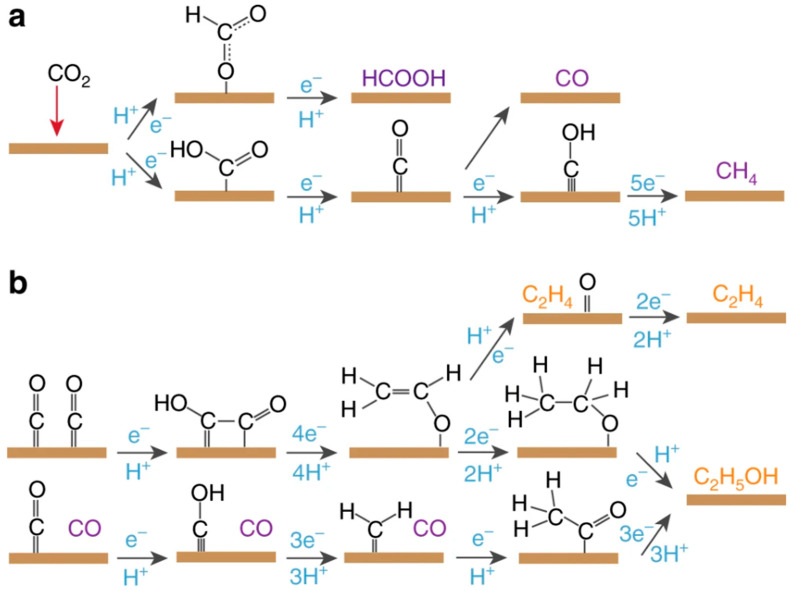
(**a**) The pathway to C_1_ products (HCOOH, CO, and CH_4_), and (**b**) the pathway to C_2_ products (C_2_H_4_ and C_2_H_5_OH), represented in purple (C_1_ molecule) and orange (C_2_ molecule). (Adapted with permission from Ref. [76], copyright 2018 Springer Nature, Ren et al.).

CO_2_ reduction poses a significant challenge due to its chemical inertness. The presence of large overpotential and low selectivity hinders the attainment of the desired products. From a materials design perspective, it is essential for reaction pathways to align with the goal of obtaining value-added products in the C1 category (formates, carbon monoxide, and methane) or C2 category (ethylene and ethanol) (Figure 8) [75,76]. When aiming for C2 product formation, the challenging task involves promoting C–C coupling while simultaneously suppressing CO release and the generation of C1 products and H_2_. The initial conversion of CO_2_ to CO is a crucial step, and C–C coupling is promoted to generate C2 products, where both the CO_2_RR and CORR exhibit similar overpotential [77,78]. The adsorption energy of CO on the catalyst surface emerges as a major factor influencing the activation of CO for C–C coupling or bond formation. If the catalyst binds the CO products too strongly or too weakly, the subsequent reduction of CO will be impeded. Ultimately, maintaining an appropriate potential window is critical for obtaining selective products [76,77,78].

Noble metal aerogels (NMAs) are considered promising catalysts due to their high conductivity, catalytic activity, abundant electron/mass transfer channels, robust structure, and plasmonic properties [79,80]. However, the fabrication of NMAs is challenging due to low gelation kinetics (taking several hours to a few weeks) and difficulties in controlling their microstructure [81,82]. The Alexander group has developed well-defined Au–Pd core–shell aerogels with a random element distribution at room temperature [83]. This achievement was made possible by adjusting the metal nucleation and growth through a disturbance-assisted dynamic shelling strategy, which is a ligand-free process enabling fabrication within 10 min. The core–shell Au–Pd aerogel demonstrates a 99.96% Faradaic efficiency (FE) for CO at −0.5 V, and an overpotential of 390 mV [83].

A two-dimensional electrode is primarily used for the CO_2_RR by employing noble metals, transition metals, and metal oxide deposition on glassy or fibrous carbon paper [84,85,86]. The substrate consists of a catalytic film fabricated using solvent evaporation methods, exhibiting low wettability and few catalytically active sites. Additionally, the low CO_2_ transport capability and sluggish access of CO_2_ and electrolytes to the catalytic active sites render the electrochemical process inefficient [26,87,88]. Choi et al. propose three-dimensional graphene-based hydrogels (GHs) as an alternative electrode, characterized by a high surface area, rational porosity, homogenized conductive pathways, and cost efficiency [74]. The incorporation of water molecules in these hydrogels enhances their wettability and facilitates electrolyte access in an aqueous medium. The graphene hydrogel, when covalently bound to cationic iron porphyrin (FePGH), forms an efficient catalytic complex that demonstrates effective CO_2_ reduction at a low overpotential. These materials exhibit an impressive Faradaic efficiency (FE) of 96.2% for CO_2_ catalysis to CO at a low overpotential of 280 mV. In this context, 3D porous hydrogels not only provide efficient conductive pathways but also facilitate effective gas diffusion at catalytic active sites [74].

The Han group utilized a Pd–Cu bimetallic aerogel, a porous, non-supported electrocatalyst, for CO_2_RR to CH_3_OH production, which offers high catalytic activity and stability [89]. This aerogel’s structure enhances performance synergistically, leveraging its network structure and the valence states of Pd and Cu, resulting in an 80% Faradaic efficiency (FE) for CH_3_OH production. This is achieved at a current density of 31.8 mAcm^−2^ and a low overpotential of 0.28 V. Its enormous porous structure and nanoparticle properties are combined and magnified through self-assembly on the macroscale [89]. The use of non-precious Mn elements in metal complexes shows promise for the reduction of CO_2_ to CO as a homogeneous catalyst. To electrocatalyze CO_2_ reduction with such homogeneous catalysts, an organic solvent medium is employed to prevent complex decomposition in the presence of water [90,91]. However, the trade-off between poor stability and low current density in organic solvents poses a critical challenge in the CO_2_RR process. Additionally, the high redox potential and environmental friendliness of MnO as a promising electrode material are hindered by its self-agglomeration, limiting the catalytic active sites, restricting electron and proton transport, and impeding electrical conductivity [92,93]. The Shi group utilized 3D nitrogen-doped graphene aerogels (NGAs) with dispersed MnO nanoparticles for CO_2_ reduction [94]. Their 3D hydrogel structure exhibits low density, excellent electrical conductivity, high specific surface area, good mass transport, and outstanding chemical stability. The homogeneously dispersed MnO in their NGA hybrid catalyst provides an interconnected microporous structure, ensuring long-term stability and a low onset potential of −0.27 V vs. RHE, achieving an 86% Faradaic efficiency (FE) at −0.82 V vs. RHE and a current density of 7.1 mA cm^−2^. N doping on the graphene aerogel limits and suppresses H_2_ production, while well-crystallized active sites of MnO contribute to cooperative and synergetic effects, resulting in excellent CO_2_RR. The MnO incorporation in the NGA decreases the CO_2_ adsorption barrier, promoting a drastic transition from *CO_2_ to *COOH [94]. 

Most catalysts struggle to meet the industrial-scale requirements for current density, which require them to exceed 100 mA cm^−2^ with a CO_2_ conversion efficiency of over 90% [95]. For instance, when single-atom metals (Fe, Co, Ni, and Cu) are doped onto N-containing carbon, the resulting catalyst demonstrates a 96.8% Fe conversion for CO_2_ to CO at −0.8 V and a current density of 27 mA cm^−2^ [96].

**Figure 9:**
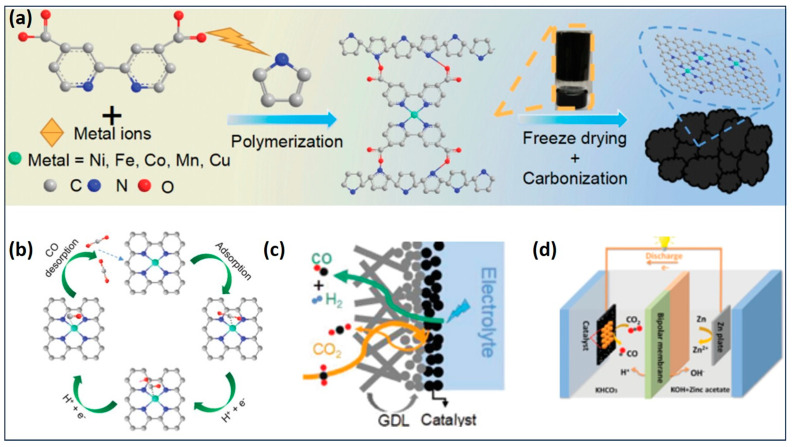
(**a**) Schematic of the preparation process for M-doped N-containing carbon aerogels; (**b**) catalytic mechanism of CO_2_RR on catalysts; (**c**) schematic configuration of a flow cell reactor with a GDL loaded with a catalyst; and (**d**) configuration of the Zn–CO_2_ battery, which comprises C paper coated with catalysts in a CO_2_-bubbled electrolyte. (Adapted with permission from Ref. [100], copyright Wiley-VCH GmbH).

In the case of Ni single atoms on N-containing carbon, this catalyst achieves a satisfactory industrial-scale current density of 213.2 mA cm^−2^ with an Fe conversion rate of 96.9% at −0.55 V for the CO_2_RR [97]. Nevertheless, there remains a desire to attain a higher conversion efficiency, specifically exceeding 300 mA cm^−2^ current density for CO_2_ reduction [98,99]. The Hou research group has developed a carbon aerogel with a 3D hierarchical crosslinked nanostructure incorporating Ni-N sites (Figure 9) [100]. This innovative catalyst exhibits a remarkable 98% Faradaic efficiency at −0.8 V, providing an industrial-level current density of 300 mA cm^−2^ for the conversion of CO_2_ to CO. The Ni-N sites play a crucial role in facilitating CO_2_ protonation, electron transfer, and the formation of *COOH, which are the rate-determining steps. Furthermore, Ni-N carbon aerogels find an application as cathodes in Zn-CO_2_ batteries, demonstrating a notable power density of 0.5 mW cm^−2^ [100].

The Ma group utilized Bi–Sn aerogel for the formation of value-added products of HCOOH from the electroreduction of CO_2_ [101]. The group produced a non-precious 3D interconnected bimetallic aerogel at ambient temperatures, which favors mass transport and provides a 90.9% FE of HCOOH. The coexistence of Bi and Sn optimizes the energy barrier for the production of HCOOH. Individually, Sn (200) and Bi (012) exhibit the strongest and lowest binding energy of *HCOO, respectively, which is not suitable for the electrocatalytic process. The Bi-Sn bimetallic combination provides a moderate adsorption energy for the intermediates and facilitates the CO_2_RR more effectively than the HER. The *COOH generation is the limiting step, with a significantly lower Gibbs free energy for the Bi-Sn aerogel (0.62 eV) compared to Bi (1.19 eV) and Sn (0.85 eV) individually [101]. The Xiang group designed a Cu–Bi aerogel for the selective formation of formates through electrocatalytic CO_2_RR. Their Cu–Bi aerogel, possessing self-supporting properties, demonstrates a 96.5% Faradaic efficiency (FE) at −0.9 V vs. RHE. This high efficiency is attributed to its ability to facilitate electron transfer and matter transport [102]. The Kraatz group developed aerogels from peptides and combined them with graphene oxide and Ag nanoparticles for electrochemical CO_2_ reduction [103]. Their addition of Ag nanoparticle catalysts to the aerogels showed a 46% FE at −0.8 V vs. RHE, and the incorporation of graphene oxide improved the electroreduction of CO_2_ to an 88% FE at −0.7 V vs. RHE. Their design perspective was to fabricate the aerogel based on a biobased material rather than an inorganic aerogel in order to reduce environmental impact, as the former is biodegradable and non-toxic [103].

**Figure 10:**
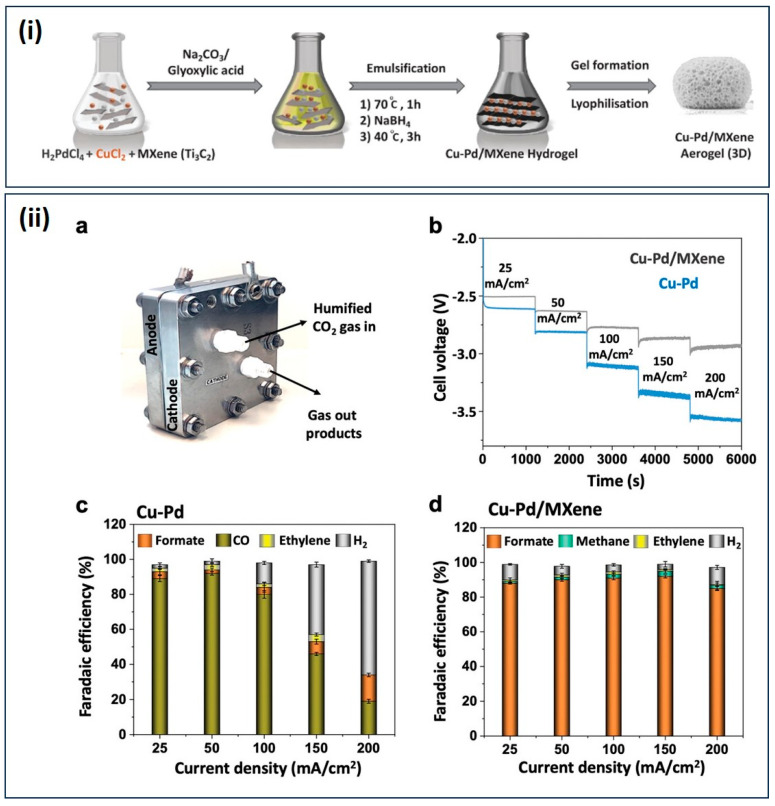
(**i**) Schematic of the synthesis of 3D Cu–Pd MXene hydrogel; (**ii**) (**a**) MEA cell for CO_2_RR, (**b**) V vs. t at different current steps in the range of 25, 50, 100, 150, and 200 mA cm^−2^, (**c**) FE of Cu–Pd, and (**d**) FE of Cu–Pd MXene aerogels in 0.5 M KOH using the MEA cell. (Adapted with permission from Ref. [116], copyright 2023 Wiley-VCH GmbH).

CO_2_-to-formate production on an industrial scale requires a suitable potential and a minimum high current density of >150 mA cm^−2^ [104]. Post-transition metal catalysts, such as Pb, Sn, and In, have the capability to selectively produce formates from CO_2_ [105,106,107]. However, these materials pose a concern, as a large overpotential is necessary to activate the CO_2_. Bimetallic catalytic engineering designs tuned intermediate energies by increasing the number of active sites for reactions. Intermediates from CO_2_ play a crucial role in maintaining regulatory effects. If intermediates bind too strongly with the catalyst, they may fail to activate the intermediate molecules for further reactions, resulting in poisoning of the materials [108,109,110]. Catalytic performance also depends on the morphology and support of the essential catalytic components. MXenes (M_n+1_X_n_T_x_, where M = transition metals, X = C or N, and Tx = surface groups such as = –O, –OH, –F, etc.) are useful for energy conversion and storage due to their 2D structure and hexagonal lattice groups, providing a large surface area, and good mechanical and electrical conductivity [111,112,113]. However, the catalytic activity sites and surface area of MXenes can be affected by the aggregation of materials due to strong van der Waals forces among the stacking layers [114]. This issue might be addressed by improving the properties of intermediates through the restacking of MXenes via doping, or through the incorporation of heteroatoms into the structure [114,115]. In the context of these considerations, Abdinejad et al. designed 2D MXene aerogels (Ti_3_C_2_T_x_) with a Cu–Pd bimetallic composition using a lyophilization process, resulting in a 3D structure (Figure 10). The 3D Cu–Pd/MXene aerogels, when employed as a membrane electrode assembly (MEA), demonstrated a formate formation efficiency of 93%, selectivities exceeding 90%, a current density of 150 mA cm^−2^, and a fuel cell energy efficiency of 47% (cell potential −2.8 V) [116]. The synergy effect between Cu and Pd in tuning the electronic structure guided the reaction towards selective product formation. The multilayered 3D structure provided a higher amount of catalytic active sites and enhanced conductivity, thereby boosting CO_2_-to-formate electroreduction [116].

### 4.2. Gel Electrocatalysts for the ORR and OER

**Figure 11:**
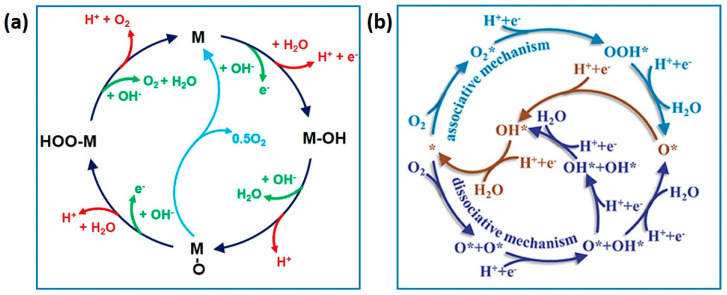
(**a**) OER mechanism (adapted with permission from Ref. [119], copyright 2018 American Chemical Society); and (**b**) ORR within associative and dissociative mechanisms (adapted with permission from Ref. [120], copyright 2019 Ma et al.).

The oxygen evolution reaction (OER) and oxygen reduction reaction (ORR) play pivotal roles in metal–air batteries, polymer electrolyte fuel cells, water electrocatalysis, and photo(electro)catalytic water splitting. The cathode reaction in an ORR is characterized by a substantial overpotential, which consequently impedes efficiency [117,118,119]. In rechargeable metal–air batteries and water electrocatalysis, the OER exhibits rate-limiting steps. The intricate sluggish kinetics associated with these reactions pose a challenge to the efficiency of energy storage and conversion devices. Understanding the reaction mechanisms of the OER and ORR is crucial for the rational design of electrocatalysts (Figure 11). This understanding is vital for optimizing the performance of applications such as metal–air batteries, fuel cells, and water splitting processes [117,118,120].

Transition metals are considered as alternative electrocatalysts due to their active properties in both the oxygen reduction reaction (ORR) and oxygen evolution reaction (OER), along with their low costs and abundance [121,122]. However, in practical applications like Zn–air batteries, they exhibit lower efficiency rates, attributed to their low conductivity. On the contrary, transition metal alloys such as NiFe, NiCo, and FeCo are recognized as efficient bifunctional oxygen electrocatalysts because they offer good conductivity [123]. These alloys, featuring dual dissimilar transition metals, induce intrinsic polarity, resulting in higher catalytic activity compared to single metallic catalyst designs. Carbon nanotubes and graphene are commonly employed as supports for the active catalyst of transition metals [124]. Nevertheless, significant challenges arise, including the aggregation of active particles and peeling off from the substrate due to poor non-intimate contact with the support, leading to reduced stability and performance under operational conditions [125,126,127]. The group led by Jong–min proposes a solution to address these challenges by fabricating an intimate contact between active particles and the conducting substrate [128]. They developed FeCo-anchored–N-doped dual-network C aerogels (FeCo/N–DNC), comprising a three-dimensional (3D) porous structure that effectively confines and immobilizes active particles on the structure. This intimate and close contact yields synergistic coupling effects, significantly enhancing the active sites and facilitating highly efficient electron transfer from the active particle to the C supports, thereby lowering the local working functions. FeCo/N–DNC is synthesized from the cyanometallate crosslinked chitosan/graphene oxide dual-crosslinked hydrogel, overcoming limitations such as leaching, separations, and the aggregation of metal alloys during operational conditions. The abundant pores and high surface area of FeCo/N–DNC provide opportunities for fast electrolyte access, large catalytic active sites, and proficient charge transfer, making it a promising cathode material for Zn–air batteries. In fact, it demonstrates a considerable power density of 115 mW cm^−2^ at a current density of 0.2 A cm^−2^ [128].

**Figure 12:**
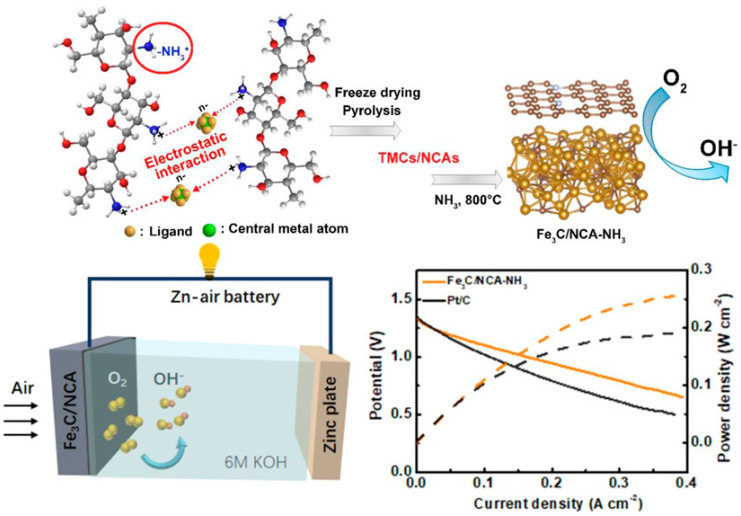
Synthesis of TMCs/N-doped C aerogels; schematic representation of a Zn–air battery; and comparison of the polarization and power density curves for Zn–air batteries assembled with Fe_3_C/N-doped C aerogels, and with 20% Pt/C. (Reprinted (adapted) with permission from ACS Appl. Mater. Interfaces 2022, 14, 22151–22160 [132], copyright 2022 American Chemical Society).

Transition metal carbides (TMCs) are emerging as alternative low-cost catalysts, replacing precious materials such as platinum (Pt), iridium (Ir), and ruthenium (Ru) in electrochemical energy conversion [129,130]. In TMCs, the broadening of the d-orbitals of transition metals induces high electrocatalytic activity on the metal sites due to the hybridization of the d-orbital metals with the s- and p-orbitals of carbon (C). Although TMCs are efficient electrocatalysts, aggregation during the catalytic process causes crystallization regrowth, ultimately degrading their catalytic efficiency [128,131]. The Liu group utilized an amino-protonated chitosan–metal complex hydrogel, combining metal carbides and N-doped C aerogels, to reduce aggregation while maintaining good electrical conductivity and resistance to corrosion (Figure 12) [132]. Furthermore, heteroatoms and defects in the C frameworks influence the redistribution of charge density on metal-based catalysts, consequently improving catalytic activity. TMCs/N-doped carbon aerogel complexes are prepared using chitosan, where the protonated –NH_3_^+^ group of chitosan forms a hydrogel with metal anions (TMCs) through electrostatic interactions. The resulting structure is highly porous, achieved through freeze-drying and pyrolysis of the complex. Fe_3_C/N-doped carbon, employed as the cathode in Zn–air batteries, exhibit a power density of 250 mW cm^−2^ (Figure 12). The N-doped (graphitic N, pyridinic N) and defective carbon modifies and redistributes the charge density of the Fe active site, accumulating on the Fe_3_C side. This accumulation decreases the free energy of the oxygen reduction reaction (ORR) and enhances the ORR’s performance.

In energy conversion systems, such as water splitting, the oxygen evolution reaction (OER) plays a crucial role, involving four electron transfer steps (4OH^−^ → O_2_ + 2H_2_O + 4e^−^) [133,134]. Transition metal oxides/hydroxides with multiple oxidation states exhibit excellent pseudocapacitive performance for both oxidation and reduction reactions in catalyzing the oxygen evolution reaction [135,136]. These materials demonstrate high theoretical activities, and their pseudocapacitance potential is often lower than the OER standard potential. However, the hydrophobic nature of most catalysts limits their performance due to poor wettability, which hinders efficient electrolyte access, resulting in poor OER catalytic activity and kinetics [137,138]. 

In addressing this limitation, O- and N-containing functional groups (–COOH, –OH, –C=O) are introduced to induce a substantial amount of hydrophilic functionality [139]. This promotes wettability through H bonding, improving electrolyte access to the catalyst. Furthermore, N doping modifies the charge density on C, enhancing the pseudocapacitive performance [137]. Liu et al. designed and synthesized 3D NiCoFe hydroxide nanoplates and a N-doped C hydrogel hybrid for OER catalytic activity [140]. Their hydrogel offers multidimensional conductive networks, while N doping ensures its high wettability, resulting in excellent pseudocapacitive performance with a specific capacitance of 1849 F g^−1^ and an energy density of 31.5 Wh Kg^−1^ [140].

Metal–organic gels (MOGs) are emerging as promising materials for electrochemical applications in energy conversion, particularly in oxygen evolution reactions (OERs) for water splitting [35,62]. The low active surface area of conventional transition metal oxides, hydroxides, nitrides, chalcogenides, and phosphatides contributes to slower kinetics and higher overpotentials. On the other hand, metal–organic frameworks (MOFs) exhibit high surface areas suitable for electrochemical applications. However, they face challenges such as stability issues, low diffusion rates, and poor conductivity of the organic sites within their structure, resulting in overall electronic conductivity limitations [141]. In contrast, MOG-based materials demonstrate efficient mass and charge transport capabilities in conversion and energy storage processes. This is attributed to the presence of abundant active defect sites on the structure, which are exposed to reactant intermediates, enhancing the overall performance of these materials [37,142]. Moi et al. prepared metal–organic gels (MOGs) containing Co(II)/Ni(II) and observed and compared their electrocatalytic performance for OERs [143]. The results indicated that fibrous Co–MOGs exhibited a lower overpotential of 312 mV at a current density of 10 mA cm^−2^ compared to globular Ni–MOGs. Their fibrous structure, with higher porosity, facilitates mass transport and allows for the maximum utilization of active sites, leading to enhanced activation [143].

**Figure 13:**
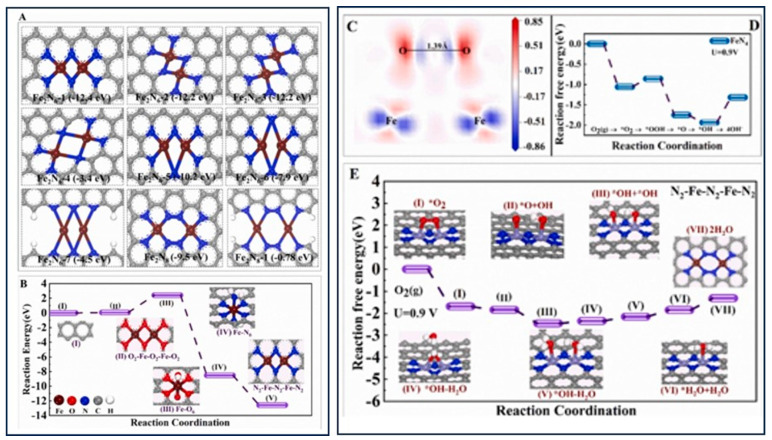
DFT-calculated (**A**) optimized potential configurations for Fe-N-C with the average coordination numbers of Fe-N and Fe-Fe being approximately 4 and 1, respectively (wine: O, blue: N, gray: C, white: H), (**B**) formation pathway from the Fe-C-O polymer to Fe-N-C at 800 °C, and (**C**) charge density difference for the O_2_ adsorption structure on the N2-Fe-N2-Fe-N2 site, where blue indicates decreasing charge density, and red indicates increasing charge density; Gibbs free energy diagrams of catalysts for (**D**) Fe-N4 and (**E**) N2-Fe-N2-Fe-N2 at 0.9 V. (Adapted with permission from Ref. [148], copyright 2022 Elsevier Ltd.).

Atomically dispersed and single-atom catalysts on heterogeneous supports are utilized for efficient energy conversion and storage technologies. Metal–organic frameworks (MOFs), covalent organic frameworks (COFs), carbon black, and zeolites are all employed to prepare single-atom dispersed active sites based on pore confinement effects [144]. However, the micropores of these materials limit mass transfer, especially in air- or oxygen-based batteries. A minimum mean free path of 60 nm or larger is required to achieve efficient mass transport phenomena [145]. It is also essential to consider maintaining the surface area and the number of active sites, which decrease due to macro-pores. A hierarchical structure combining macro-/meso-/micro-pores could provide simultaneous mass transport and surface area on the catalysts. Carbon aerogels, based on assembly or pyrolyzation, could offer hierarchically porous and atomically dispersed active centers by utilizing sol–gel technology [59,146,147]. The Feng group has developed a metal-doped polymer aerogel in which hierarchical porous carbon aerogels are dispersed with Co, Ni, and Fe atoms, providing a dual-metal N_4_ catalytic active center (Figure 13) [148]. The Co-N4 catalyst demonstrates catalytic activity for the OER and ORR in an alkaline medium. The Ni-N4 exhibits catalytic activity for the reduction of CO_2_. The Fe-N_4_ carbon aerogels exhibit a remarkable maximum power density of 167 mW cm^−2^ and an energy density of 956 Wh kg^−1^ when used as an air electrode for a solid-state Zn–air battery. The Fe-N_4_ catalyst facilitates an efficient ORR due to its hierarchical pore morphology, enabling fast mass transport and high catalytic activity. The high catalytic activity of the ORR was evaluated, and the rate-determining steps were identified as the reduction of *OH and H_2_O desorption (Figure 13). H_2_O molecules are adsorbed on one metal site, while the other metal site provides the opportunity for further ORRs in N2–Fe–N2–Fe–N2 carbon aerogels compared to the classical Fe–N4. Additionally, the formation of H_2_O_2_ is hindered, as the key OOH intermediate is absent. The bimetallic active sites in the N2–Fe–N2–Fe–N2 carbon aerogel exhibit a synergistic effect, accumulating more electrons on the O–O antibonding orbital. The donation of more electrons to the O–O antibonding orbital results in a longer bond length of 1.39 Å compared to the O2 bond length of 1.2 Å. The dual Fe atoms in the N2–Fe–N2–Fe–N2 carbon aerogel enhance electron transfer to the O–O, increasing the interaction between the Fe and O. This causes the localization of electrons on the oxygen atoms and reduces the bonding electrons between O and O, promoting O–O dissociation. Ultimately, this leads to excellent catalytic activity in the ORR [148].

**Figure 14:**
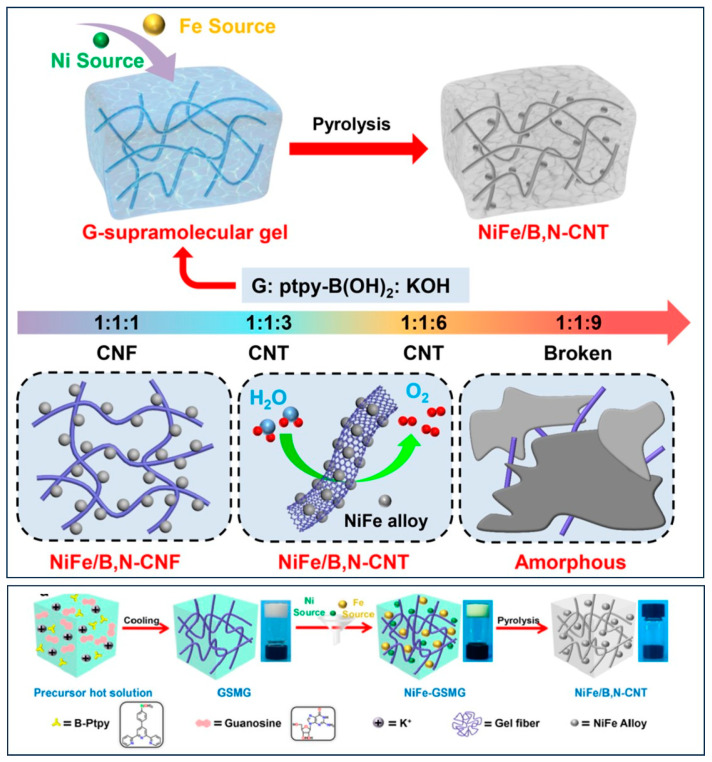
Diagram depicting the adjustable synthesis and structural modification of supramolecular gel-derived active materials, involving varying KOH concentrations in the precursors. This schematic outlines the synthesis process for NiFe/B, N-CNT obtained from gel-derived GSMG. (Adapted with permission from Ref. [160], copyright 2022 Elsevier Ltd.).

Nanostructured supramolecular gels, formed through self-assembled fibrillar networks via non-covalent interactions, represent promising soft functional materials for energy conversion and storage applications [149,150,151]. Their appeal lies in their tunable structure, modifiable compositions, and controlled functionality. The 3D network of these supramolecular gels establishes a hierarchical structure with a high surface area, serving as a conductive structural chassis for efficient electron transfer and promoting effective electrochemical processes. However, the structural stability of monotonous fibrous materials poses a significant challenge for practical applications [152,153,154]. To address this, carbon nanotubes (CNTs) fabricated with metal components (M/CNTs) are considered effective energy materials, offering both high functional activity and structural stability [155,156]. Their functional inner cavity of hollow nanostructures, loaded with metal nanoparticles, proves suitable for energy conversion and storage [157]. The conventional methods for designing and synthesizing M/CNTs, such as chemical vapor deposition, wet chemical processes, arc discharge, and thermal decomposition, lack flexibility for precursor modifications and regulations [158,159]. They often require high energy consumption under extreme synthesis conditions, leading to impurities in the final products. The Liu group has developed a synthetic strategy to prepare B, N-doped CNTs loaded with NiFe alloy nanoparticles [160]. This is achieved through a facile self-templated conversion of guanosine base supramolecular gel, termed as GSMG. The combined effects of hollow CNTs of high conductivity and the highly functional and catalytic active sites of NiFe alloys contribute to high electrocatalytic performance in the OER process (Figure 14). NiFe/B, N-CNT-6 shows a lower overpotential of 355 mV at a current density of 10 mA cm^−2^ than the recently developed cooperative catalytic catalyst of CoO_x_ clusters in the lattice of rutile TiO_2_ (overpotential 400 mV at 10 mA cm^−2^) [160,161].

### 4.3. Gel Electrocatalysts for H_2_ Production

The internal structure of chloroplasts in plants, along with its integrated functions and high water content, has inspired the design of soft hydrogel materials for H_2_ production under light illuminations. These soft materials offer advantages over the use of large volumes of liquids, dispersions of expensive or toxic inorganic particles, or complex devices [162]. Weingarten et al. developed supramolecular hydrogels that incorporate light-absorbing chromophores (perylene monoimide amphiphile), serving as a foundation for solar light-driven hydrogen production. By carefully controlling the conditions—such as maintaining a high concentration and employing electrostatic screening—a charged supramolecular structure can be generated. This structure forms a three-dimensional (3D) gel network that readily accommodates hydration and hosts soluble components (Ni-based components), facilitating the production of solar fuels [162]. The 3D network, characterized by its ability to absorb solar light through conjugated molecules formed by a π–orbital overlap, efficiently splits excitons and transports charges to the catalytic center during the reaction process. The developed catalytic scaffold provides a model for integrated chemical systems, showcasing potential applications in the production of solar fuels and chemical transformations [162].

## 5. Summary and Outlook for Future Research

Gel materials are recognized as potential and promising electrocatalysts, primarily owing to their favorable electrochemical performance in a wide range of applications related to energy conversion and storage devices (Table 1). Their utilization continues to expand, driven by attributes such as a higher surface area, three-dimensional structural nature, increased porosity, enhanced mass flow, and improved electrical conductivity. These properties afford active catalytic sites on the structure, making gel materials highly effective in catalyzing electrochemical reactions. Moreover, there is a significant opportunity for fine-tuning the active functional groups within the structure, providing a versatile platform for further optimization of their electrocatalytic properties.

Materials design and engineering, from a structural perspective, underscores the critical significance of parameters such as size, facets, and dimensions. Within the synthesis process, steering the structural facets assumes paramount importance, particularly in the context of fostering a selective and efficient catalytic reaction process. Notably, edge sites emerge as pivotal in facilitating the CO_2_ reduction reaction (CO_2_RR). In contrast, in the CO_2_RR process, the mitigation of corner sites becomes imperative, as these sites tend to favor competitive reactions associated with the hydrogen evolution reaction (HER). Adopting strategies to diminish corner sites is therefore essential for optimizing catalytic efficiency and selectivity in CO_2_ reduction processes.

The importance of higher surface areas and increased porosity in gel materials is associated with elevated catalytic activity. However, the significance of the hierarchical structure and the specific pore size range has not yet been fully elucidated. It is crucial to investigate these aspects, as they play a pivotal role in facilitating an efficient mass flow of gases or transporting intermediate reactants. This consideration becomes particularly relevant when accounting for internal pressures within the ultra-/micro-/meso-/macro-pore system. Therefore, a comprehensive understanding of the hierarchical structure and the appropriate pore size range is essential for optimizing catalytic performance in gel materials.

Researchers emphasize that gels play a crucial role in reducing environmental impacts and mitigating the dilution of toxic metals in various applications. However, it is imperative to adopt a comprehensive strategy for effectively removing these toxic components from the composite material. This becomes especially significant in addressing the potential negative environmental impacts associated with aerogels.

Supercritical drying is deemed expensive and poses safety risks, necessitating the exploration and development of alternative processing methods. There is a growing need to consider such alternatives. One potential avenue is the investigation of alternative surfactant molecules that can yield a porous structure. This can be achieved using conventional solvent extraction instead of relying on the more complex and potentially hazardous supercritical extraction process.

It is well-established that transition or post-transition metal catalysts offer selective catalytic intermediates, particularly in situations where large overpotential is a concern, and challenges such as poisoning via intermediates or poor wettability could impede efficient catalytic activity (Table 2). Conversely, a bimetallic catalytic design strategy has the potential to increase the number of active sites, modifying or optimizing the adsorption–desorption energy or energy barrier of intermediates. The choice of utilizing noble, transition, non-transition, or a mixed combination of metallic types can be based on considerations of cost reduction and electrocatalytic performance perspectives. Moreover, single-atom-doped carbon gels have demonstrated their capability to provide industrial-level current densities. Additionally, carbon-based heteroatom-doped gels offer sufficient porosity and intimate contact, facilitating localized interactions between active particles and conductive components for efficient mass and charge transfer. The broadened d-orbitals of transition metal carbides (TMCs) induce high electrocatalytic activity, yet issues such as crystallization growth or agglomeration of TMCs can lead to degradation in their catalytic activity. Combining heteroatom-doped carbon materials with the accumulation of TMCs and redistribution of charge density at transition metal active sites can decrease the free energy of both the oxygen reduction reaction (ORR) and oxygen evolution reaction (OER). For transition metal oxides or hydroxides, wettability criteria need to be considered to ensure the proper accessibility of electrolytes, although a gel-functionalized structural strategy provides opportunities for localized electrolyte accessibility. Nanostructured supramolecular gel-based chassis further offer the potential for functionalization with a hierarchical structure, enhancing effective gas, mass, and charge diffusion, and improving wettability.

However, it is essential to acknowledge that each type of gel-based catalyst, despite its individual advantages, faces specific challenges such as structural stability, uniform scale-up for production, agglomeration and leaching, intermediate poisoning, and selectivity. In the pursuit of finding a universally efficient electrocatalyst for respective applications, materials engineers need to consider all aspects of the materials’ challenges and strengths, strategically combining the categorized materials’ strengths at the initial state.

In conclusion, gel materials show promise as alternatives for advanced energy conversion and storage. Their appeal lies in tunable properties, hierarchical porous frameworks, self-catalysis, and cost-effectiveness. Other notable attributes include lower processing temperatures, excellent porosity, chemical stability, and eco-friendly layer-by-layer film deposition. The intrinsic stretchability, flexibility, and bending abilities preserve high charge flexibility. Three-dimensional polymer gels efficiently accommodate volume expansion, making them ideal for managing ion movements during electrochemical processes. Overall, the diverse and advantageous properties of gel materials highlight their significant potential in advancing energy technologies.

## Figures and Tables

**Table 1:** Performance comparison of gel-based electrocatalysts for the OER, ORR, and CO_2_RR.

Applications	Materials	Performance	Ref.
Zn–air battery (ORR)	Fe_3_C/N-doped C aerogels	P 253 mW cm^−2^ at current density 0.4 A cm^−2^, E_on_ 0.93 V, Tafel slope 76 mV dec^−1^, specific capacity 820 mA h g^−1^	[132]
Zn–air battery (ORR)	20% Pt/C	P 190 mW cm^−2^ at current density 0.4 A cm^−2^, Tafel slope 75 mV dec^−1^, specific capacity 742 mA h g^−1^, η 0.39 V at current density 10 mA cm^−2^	[132]
Zn–air battery (ORR)	FeCo/N-DNC	P 115 mW cm^−2^ at current density 0.2 A cm^−2^, E_on_ 0.89 V, E_1/2_ 0.81 V, specific capacity 804 mA h g^−1^, E 988 W h Kg^−1^ at 5 mA cm^−2^	[128]
Zn–air battery (ORR)	Pt/C + RuO_2_	P 109 mW cm^−2^ at current density 0.15 A cm^−2^, E_on_ 0.98 V, E_1/2_ 0.84 V, specific capacity 699 mA h g^−1^, E 870 W h Kg^−1^ at 5 mA cm^−2^	[128]
Zn–air battery (ORR)	Fe–N_4_ carbon aerogel	P 167 mW cm^−2^ at current density 0.22 A cm^−2^, E 956 Wh kg^−1^, E_1/2_ 0.93 V, Tafel slope 53 mV dec^−1^, C_dl_ 9.3 mF cm^−2^	[148]
Zn–air battery (ORR)	FeCo/Co_2_P/Fe_2_P-N-doped C aerogel	P 174 mW cm^−2^ at current density 0.3 A cm^−2^, E E_on_ 0.88 V, E_1/2_ 0.79 V, Tafel slope 117 mV dec^−1^, specific capacity 730 mA h g^−1^, E 956 W h Kg^−1^ at 5 mA cm^−2^	[163]
ORR	Pd_3_Cu aerogel	Limiting current density 5.8 mA cm^−2^, E_1/2_ 0.9 V, C_dl_ 8 mF cm^−2^, TOF 500 s^−1^ at 0.85 V	[164]
ORR	Organic xerogel (Fe-N-C)	E_onset_ 0.73–0.76 V, E_1/2_ 0.49–0.54 V, J_d_–−2.88 to −3.88 mA cm^−2^, Tafel slope 70–93 mV dec^−1^	[165]
ORR	Pd_3_CuFe_0.5_ aerogels	E_1/2_ 0.92 V, limiting current density −7.6 mA cm^−2^, C_dl_ 21.9 mF cm^−2^, Tafel slope 96 mV dec^−1^	[166]
Zn–CO_2_ battery	Ni-N-containing carbon aerogel	P 0.5 mW cm^−2^ at current density 3 mA cm^−2^, Tafel slope 86.6 mV dec^−1^, FE_CO_ 91% (300 mA cm^−2^)	[100]
OER	NiCoFe hydroxide nanoplates/N–doped C hydrogel hybrid	E 31.5 Wh Kg^−1^ at specific capacitance 1849 F g^−1^, Tafel slope 31 mV dec^−1^, η 250 mV (j = 10 mA cm^−2^), TOF 0.17 s^−1^, C_dl_ 34.9 mF cm^−2^	[140]
OER	FeCo/Co_2_P/Fe_2_P-N-doped C aerogel	η 281 mV at current density 10 mA cm^−2^, Tafel slope 85 mV dec^−1^, ECSA 353.1 m^2^ g^−1^, C_dl_ 8.6 mF cm^−2^	[163]
OER	Co–MOGs	η 312 mV at current density 10 mA cm^−2^, Tafel slope 84 mV dec^−1^	[143]
OER	Ni–MOGs	η 418 mV at current density 10 mA cm^−2^, Tafel slope 107 mV dec^−1^	[143]
OER	NiFe/B, N-CNT	η 355 mV at current density 10 mA cm^−2^, Tafel slope 116.2 mV dec^−1^, C_dl_ 11.9 mF cm^−2^	[160]
OER	Co/TiO_2_	η 400 mV at current density 10 mA cm^−2^, Tafel slope 65 mV dec^−1^, catalyst surface area (j*_BET_*) 2.04 mA cm^−2^ at 1.65 V, C_dl_ 0.26 mF cm^−2^, TOF 3.2 s^−1^ at 350 mV	[161]
OER	Fe_x_Co_y_La_z_ aerogel	η_onset_ 201 mV, η_10_ 209 mV, η_100_ 319 mV, Tafel slope 49.8 mV dec^−1^, mass activity 505.4 A g_aerogel_^−1^ at 1.63 V (vs. RHE)	[167]
OER	FeNi–O aerogel	η 280 mV at current density 50 mA cm^−2^, Tafel slope 3.25 mV dec^−1^, ECSA 148.5 cm^2^ g^−1^, C_dl_ 5.9 mF cm^−2^	[168]
HER	Pd–Co@Pd NPs–NF (MOGs)	η 57 mV at current density 10 mA cm^−2^, Tafel slope 55 mV dec^−1^	[62]
HER	Pt_0.25_Co aerogel	η 23 mV at current density 10 mA cm^−2^, Tafel slope 28 mV dec^−1^, C_dl_ 4.8 mF cm^−2^, ECSA 152.4 m^2^ g^−1^, TOF 63 s^−1^ at 0.1 V, R_ac_ 2.13 Ω cm^−2^, R_d_ 1.0 cm^−2^	[169]
CO_2_RR	Porphyrin-based graphene hydrogel (FePGH)	CO FE 96.2%, η 280 mV at current density 0.42 mA cm^−2^, TOF 0.8 s^−1^, Tafel slope 118 mV dec^−1^	[74]
CO_2_RR	Pd–Cu aerogels	CH_3_OH FE 80%, at current density 31.8 mA cm^−2^, η 0.24 V, Tafel slope 124.4 mV dec^−1^	[89]
CO_2_RR	Ni-N-containing carbon aerogel	CO FE 98%, at −0.8 V, current density 300 mA cm^−2^, Tafel slope 86.6 mV dec^−1^, C_dl_ 9.3 mF cm^−2^	[100]
CO_2_RR	CoPc@-N-C aerogel	CO FE 92.4%, at −0.8 V, current density 21.7 mA cm^−2^, TOF 1.23 s^−1^, Tafel slope 188 mV dec^−1^, C_dl_ 8.4 mF cm^−2^, ECSA 211.5 cm^2^	[170]
CO_2_RR	Bi–Sn aerogel	HCOOH FE 93.9%, at −1.0 V, current density 9.3 mA cm^−2^, C_dl_ 2.14 mF cm^−2^	[101]
CO_2_RR	Au-Pd core–shell aerogel	CO FE 99.9%, at −0.5 V, η 390 mV, Tafel slope 182 mV dec^−1^	[83]
CO_2_RR	Cu–Bi aerogel	CO FE 96.5%, at −0.9 V, C_dl_ 0.22 mF cm^−2^	[102]
CO_2_RR	MA-FF-GoX-Ag	CO FE 88%, at −0.7 V	[103]
CO_2_RR	Cu–Pd/MXene aerogels	formate FE 93%, *j*_formate_: 150 mA cm^−2^, Tafel slope 182 mV dec^−1^, ECSA 0.18 cm^2^	[116]
CO_2_RR	Cu_95_Sn_5_ aerogels	CO FE 93% with 6.58 mA cm^−2^ current density (−0.9 vs. RHE)	[171]
Li–CO_2_ battery (CO_2_RR)	N,O-diatomic dopants graphene C aerogels	Initial energy efficiency 78.4%, discharge areal capacity 18.6 mAh cm^−2^ at 20 A cm^−2^	[172]
Sensing	ZnO-doped C aerogel	Adsorption capacity 39 mg g^−1^ (crystal violet pigment), capacitance 164.7 F g^−1^, glucose sensing	[173]
Supercapacitors	C aerogels	Specific capacitance 138 F g^−1^, E 10 W h Kg^−1^, P 181 W Kg^−1^	[174]

Overpotential, η; Power density, P; Energy density, E; Reversible hydrogen electrode, RHE; Double-layer capacitance, C_dl_; Electrochemical surface area, ECSA; Turn-over frequency, TOF; Activation resistance, R_ac_; Mass transport resistance, R_d_; Onset potential, E_on_; Half-wave potential, E_1/2_; Faradic efficiency, FE.

**Table 2:** Categorized gel catalysts for electrocatalytic applications.

Types	Example	Advantages	Challenges
Transition metal oxides/OH/complex gels	Fe porphyrin, MnO	Multiple oxidation states, pseudocapacitance, high theoretical activities, energy storage	Poor wettability, self-agglomeration, poisoning via intermediates
Noble metal gels	Au, Ru, Ir, Pd, Au-Pd	High conductivity, abundant electron/mass transfer channels, robust structure, plasmonic properties	Low gelation kinetics, difficulty in controlling microstructure, high cost
Bimetallic gels	Noble–noble metal alloys (Au–Pd)	Mass transport facilitation, moderate adsorption energies, optimized energy barrier, selective catalytic activities, synergetic effects, cost-effective with noble metals, higher intrinsic polarity	Metal interaction understanding, reproducibility concerns, stability and agglomeration, homogeneous alloying, precise control, scale-up considerations
	Noble–transition metal alloys (Pd–Cu)		
	Transition–transition metal alloys (NiFe, NiCo, FeCoRu)		
	Non-transition metal alloys (Bi–Sn)		
	Transition–non-transition metal alloys (Cu–Bi)		
Carbon/graphene with heteroatom-doped gels	FeCo-N-dual-network carbon, Ag-GO	High surface area, rational porosity, homogenized conductive pathways, cost efficiency, chemical stability, hierarchical structure, low onset potential, dual network Improved intimate contact between active and conducting components, high wettability, lowers local working function, single-atom active sites	Exposure to extreme conditions such as high current densities or extended operation times lead to structural degradations, competing reactions may affect selectivity
Single-atom-doped C gels	(Fe, Co, Ni, Cu)-doped N-containing carbon	Provides industrial level current density over 100 mA cm^−2^	Optimization of different reaction conditions is challenging for designing a universal catalyst, scaling-up production, aggregation or leaching, catalyst poisoning
Transition metal carbide gels	Fe_3_C	Broadening d-orbitals induces high catalytic activity	Aggregation causes crystallization growth
Metal–organic gels	Co/Ni-containing organic gels	Efficient mass and charge transport, abundant active defect sites	Potential metal ion leaching within organic gel, temperature sensitivity
Nanostructured supramolecular gels	Guanosine-based supramolecular gels	Increased wettability, improved access to electrolytes, soft functional materials, tunable functionalization, composition, hydrophilic features, 3D network, hierarchical structure, efficient electron transfer chassis, effective gas diffusion	Structural stability under repeated cycle

## Data Availability

Not applicable.
